# Revision Repair of a Failed Bucket-Handle Medial Meniscal Tear Using Autologous Fibrin Clot Augmentation: A Case Report With One-Year Follow-Up

**DOI:** 10.7759/cureus.111601

**Published:** 2026-06-27

**Authors:** Mohammed Lamzira, Khatab Zakaria, Achraf Tebbaa El Hassali, Najib Abdeljaouad, Hicham Yacoubi

**Affiliations:** 1 Department of Orthopaedic Surgery, Centre Hospitalier Universitaire Mohammed VI, Oujda, MAR; 2 Department of Orthopaedics and Traumatology, Centre Hospitalier Universitaire Mohammed VI, Oujda, MAR; 3 Department of Orthopaedics and Traumatology, Mohammed VI University Hospital, Oujda, MAR; 4 Faculty of Medicine and Pharmacy, Mohammed First University, Oujda, MAR

**Keywords:** biological augmentation, case report, fibrin clot, knee arthroscopy, meniscal preservation, meniscal repair, revision meniscal repair

## Abstract

Meniscal preservation is increasingly favored because of the essential role of the meniscus in maintaining knee function and preventing early osteoarthritis. However, failure of primary meniscal repair remains a challenging clinical situation, and optimal revision strategies are not clearly established. Fibrin clot augmentation has been explored as a potential technique to enhance healing in revision meniscal repair. We report the case of a 30-year-old male who developed recurrent symptoms after arthroscopic repair of a bucket-handle tear of the medial meniscus. The patient initially sustained a twisting injury to the left knee and underwent arthroscopic meniscal repair in March 2025. Despite postoperative rehabilitation, he developed recurrent pain and mechanical symptoms in June 2025. Magnetic resonance imaging performed the same month suggested failure of the initial repair. Revision arthroscopy was subsequently performed on June 21, 2025, and revealed a persistent bucket-handle tear of the medial meniscus located in the red-white zone, consistent with failure of the previous repair. After refreshing the tear edges, an autologous fibrin clot prepared from 30 mL of peripheral venous blood was introduced arthroscopically into the lesion site. Meniscal repair was then completed using five outside-in sutures. At one-year follow-up, the patient reported complete resolution of pain, absence of locking episodes and instability, and successful return to work and sports activities. Clinical examination demonstrated a stable knee with full range of motion. The postoperative Lysholm score was 100/100, indicating an excellent functional outcome. This case highlights the potential value of autologous fibrin clot augmentation in revision meniscal repair and supports meniscal preservation as a viable treatment option after failure of primary repair of a bucket-handle medial meniscal tear. An excellent clinical outcome was observed at one-year follow-up, although structural healing could not be confirmed because postoperative MRI or second-look arthroscopy was not performed.

## Introduction

The menisci play a crucial role in knee biomechanics by contributing to load distribution, shock absorption, joint stability, lubrication, and cartilage protection [1,2]. Consequently, meniscal preservation has become a major objective in modern orthopedic surgery, particularly in young and active patients. Compared with meniscectomy, meniscal repair has been associated with better long-term functional outcomes and a reduced risk of osteoarthritis progression [3,4].

Despite advances in arthroscopic techniques and rehabilitation protocols, failure after primary meniscal repair remains a recognized challenge, with reported failure rates ranging from 10% to 25% depending on tear characteristics, patient factors, and duration of follow-up [3]. Management of failed meniscal repair is not standardized and may include partial meniscectomy, repeat repair, or biologic augmentation strategies aimed at enhancing healing potential [4].

In recent years, biological augmentation techniques have gained increasing attention in meniscal surgery [5-8]. Among these methods, autologous fibrin clot augmentation represents a simple, inexpensive, and readily available option. Fibrin clots contain growth factors and cellular components that may promote tissue healing and improve vascularization, particularly in tears involving poorly vascularized regions of the meniscus [5,6].

Although favorable outcomes have been reported for biologically augmented primary meniscal repairs, evidence regarding the use of fibrin clot augmentation in revision meniscal repair remains limited [7-11]. Therefore, reporting such cases may contribute to the growing body of literature exploring meniscal preservation strategies after failure of primary repair.

Bucket-handle tears represent a specific subtype of longitudinal meniscal tears that frequently produce mechanical symptoms and locking episodes. Because these lesions commonly occur in young and active individuals, meniscal preservation through repair is generally recommended whenever feasible. Nevertheless, failure of primary repair may still occur, creating a challenging clinical scenario in which revision repair may offer an alternative to meniscectomy while preserving meniscal tissue and knee biomechanics.

We present the case of a 30-year-old male who underwent revision arthroscopic repair of a failed bucket-handle tear of the medial meniscus using autologous fibrin clot augmentation. At one-year follow-up, the patient achieved complete symptom resolution, return to work and sports activities, and an excellent functional outcome.

## Case presentation

A 30-year-old male, a chronic smoker with a 7.5-pack-year smoking history (approximately 10 cigarettes per day for 15 years), presented with recurrent left knee pain following a previous arthroscopic meniscal repair. The initial injury occurred in September 2024 during a squatting movement at the gym, resulting in left knee pain without locking or hemarthrosis. One week later, he sustained a twisting injury while getting out of a car, associated with a transient locking sensation. Ten days afterward, he experienced sudden pain and a true locking episode while rising from a seated position.

Initial magnetic resonance imaging demonstrated a bucket-handle tear of the medial meniscus with displacement of the meniscal fragment into the intercondylar notch. Arthroscopic meniscal repair was performed in March 2025 at another institution. Following surgery, the patient completed a rehabilitation program consisting of 24 physiotherapy sessions. The patient was managed with a hinged knee brace for six weeks. Range of motion was restricted to 0°-90°, and partial weight-bearing with crutches was permitted for four weeks, followed by progressive advancement to full weight-bearing. Progressive muscle strengthening and functional rehabilitation were initiated after six weeks.

Three months after the index procedure, the patient developed recurrent medial knee pain without any new traumatic event. Clinical examination revealed tenderness along the medial joint line, positive McMurray, Thessaly, and Apley grinding tests, and mild quadriceps atrophy of approximately 1 cm. Knee range of motion was 0° to 130°. Ligamentous examination was normal, with a firm endpoint on Lachman testing and no evidence of coronal or sagittal instability.

Postoperative magnetic resonance imaging obtained after symptom recurrence demonstrated persistent abnormalities suggestive of failed healing of the repaired bucket-handle tear (Figures 1, 2).

**Figure 1:**
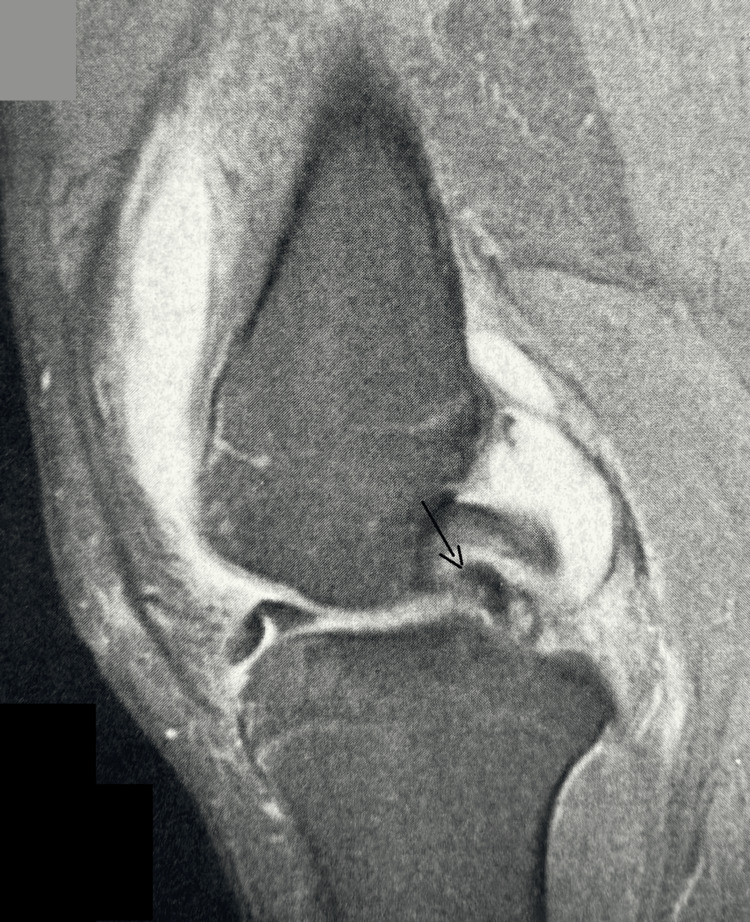
Postoperative MRI (Sagittal View) Demonstrating Persistent Findings Suggestive of Failed Healing After Primary Repair of a Bucket-Handle Medial Meniscal Tear The black arrow indicates the persistent postoperative meniscal abnormality suggestive of failed healing.

**Figure 2:**
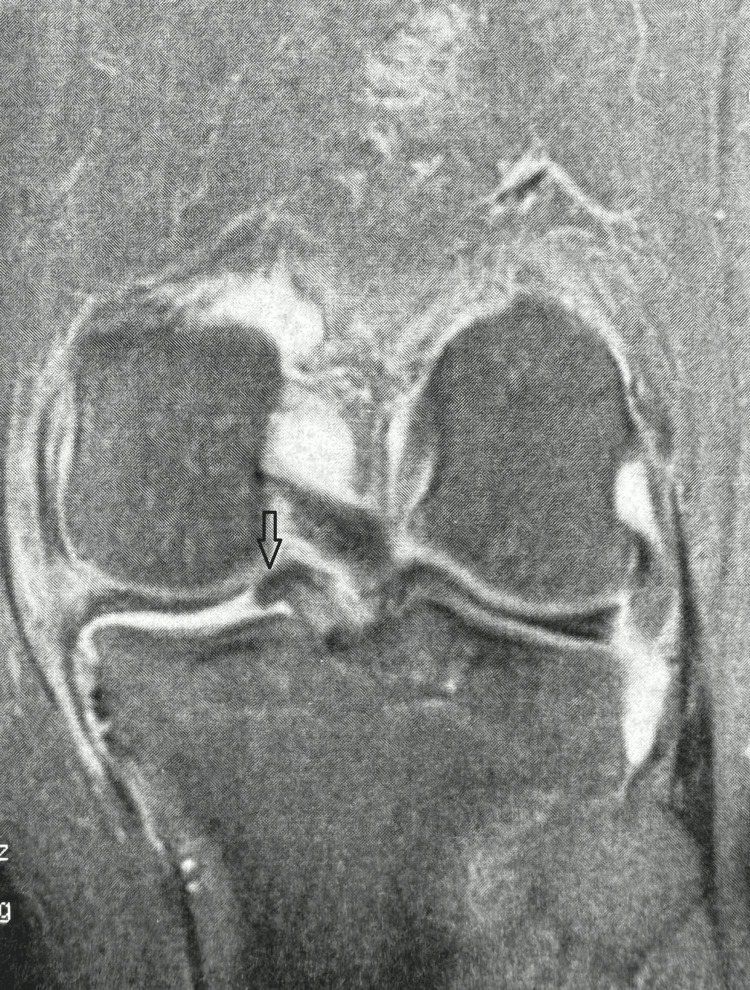
Postoperative MRI (Coronal View) Demonstrating Persistent Findings Suggestive of Failed Healing After Primary Repair of a Bucket-Handle Medial Meniscal Tear The black arrow indicates the persistent meniscal lesion on coronal MRI.

Although postoperative MRI interpretation following meniscal repair may be challenging, the diagnosis of repair failure in this case was based on the combination of recurrent clinical symptoms, MRI findings suggestive of a persistent tear extending to the articular surface, and subsequent arthroscopic confirmation during revision surgery.

Revision arthroscopy was performed using standard anterolateral and anteromedial portals under tourniquet control. Arthroscopic evaluation confirmed a persistent reducible bucket-handle tear of the medial meniscus involving the body and posterior horn and located within the red-white zone, with good tissue quality (Figure 3). The articular cartilage was intact, and both the anterior and posterior cruciate ligaments were normal. These findings supported revision meniscal repair and meniscal preservation.

**Figure 3:**
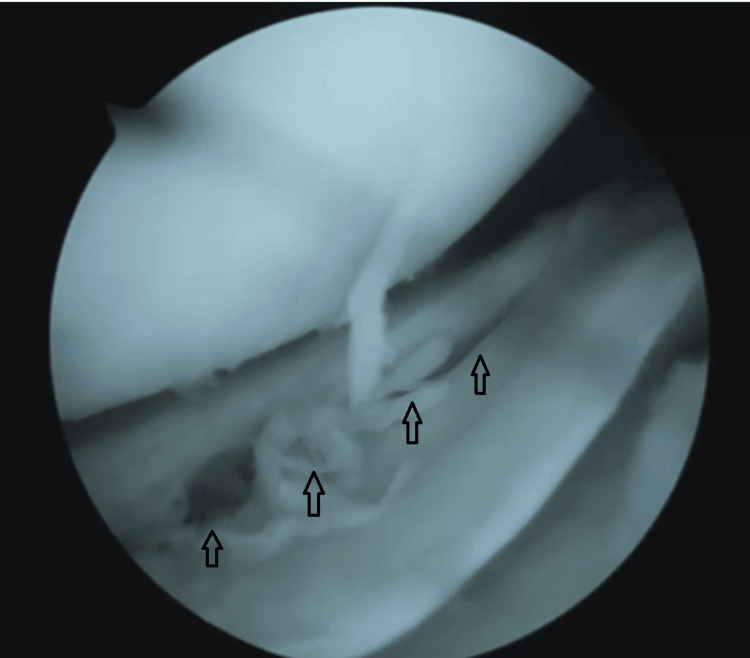
Arthroscopic Identification of the Persistent Bucket-Handle Medial Meniscal Tear During Revision Surgery The black arrows indicate the persistent bucket-handle tear identified during revision arthroscopy.

After refreshing the tear edges, 30 mL of peripheral venous blood was collected in a syringe without anticoagulant under sterile conditions. Manual stirring was performed using sterile forceps to promote clot formation (Figure 4). After approximately 15 minutes, a fibrin clot was obtained and gently compressed with sterile gauze to create a more compact structure and facilitate handling.

**Figure 4:**
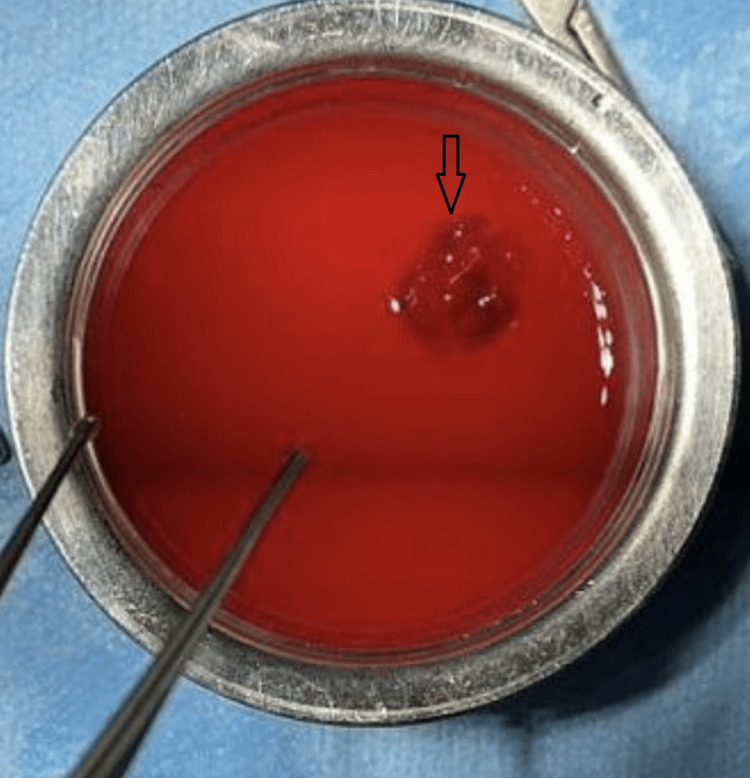
Preparation of the Autologous Fibrin Clot for Biological Augmentation The black arrow indicates the autologous fibrin clot obtained from 30 mL of peripheral venous blood and prepared intraoperatively for biological augmentation of revision meniscal repair.

A 3-0 nylon suture was then passed through both ends of the clot. The clot was subsequently introduced arthroscopically into the meniscal defect using an arthroscopic grasper and guided by the attached suture (Figure 5).

**Figure 5:**
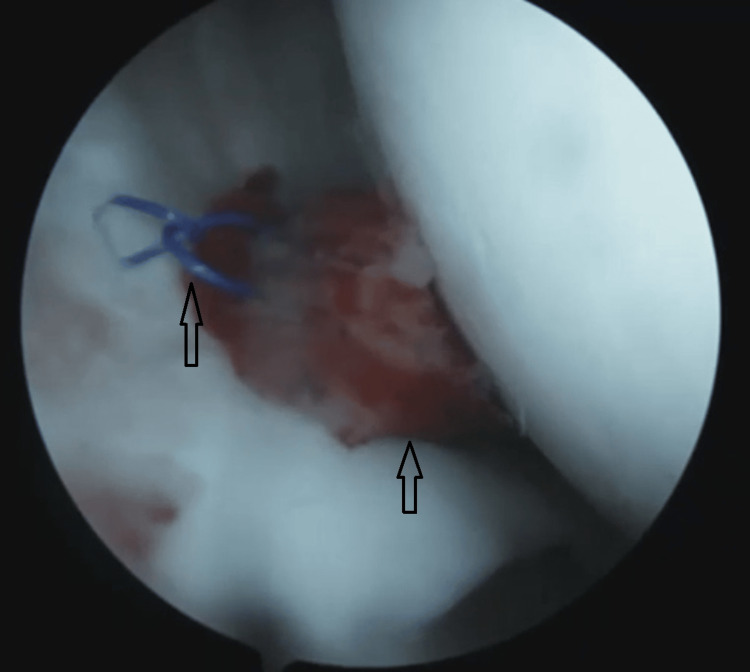
Arthroscopic Placement of the Autologous Fibrin Clot Within the Meniscal Defect The black arrows indicate the fibrin clot positioned within the meniscal defect.

During revision arthroscopy, the previously placed sutures were found to be ruptured. Meniscal repair was then completed using five outside-in No. 1 nylon sutures placed approximately 7 mm apart to ensure stable fixation of the tear (Figure 6). The procedure was performed under spinal anesthesia with the patient in the supine position and the knee flexed at 90°.

**Figure 6:**
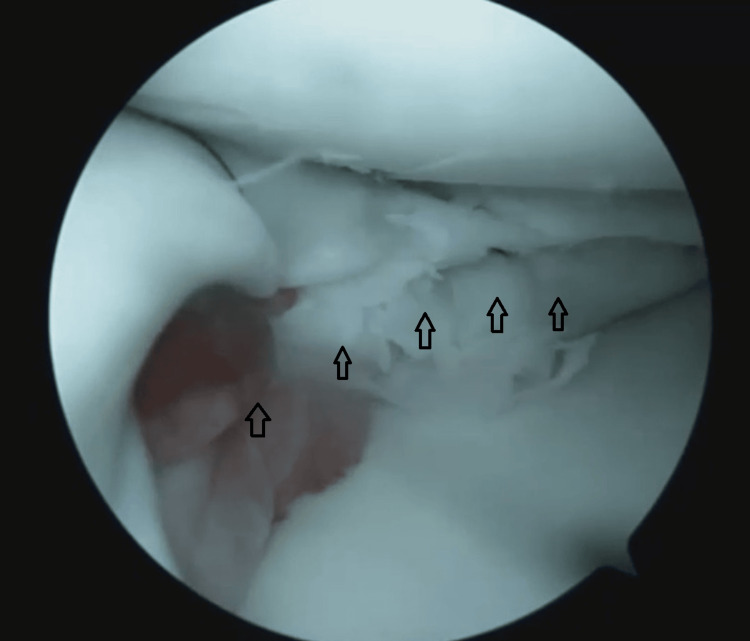
Final Arthroscopic Appearance Following Revision Meniscal Repair and Fibrin Clot Augmentation The black arrows indicate the repaired meniscal tear after fixation with five outside-in sutures.

Following revision meniscal repair with fibrin clot augmentation, a protective rehabilitation protocol was implemented. A hinged knee brace was maintained for six weeks, with knee flexion limited to 90°. Partial weight-bearing with crutches was allowed for six weeks before progression to full weight-bearing. Progressive muscle strengthening and functional rehabilitation were subsequently initiated. Running was permitted at approximately four months postoperatively, and return to sports activities was authorized at six months following revision surgery after satisfactory clinical recovery.

The postoperative course was uneventful. The knee was protected with a hinged brace according to the rehabilitation protocol, and postoperative pain was adequately controlled with standard analgesic medication. The patient was closely monitored for wound complications, infection, stiffness, thromboembolic events, and recurrent mechanical symptoms. Wound healing was satisfactory, with no signs of infection or neurovascular complications. At early follow-up visits, the patient demonstrated progressive improvement in pain and knee function without recurrent locking episodes.

At one-year follow-up, the patient reported complete resolution of pain, absence of locking episodes, and no subjective instability. He successfully returned to his professional activities and recreational sports, including running and gym-based fitness training. Return to sports was gradual and began approximately six months after revision surgery. At final follow-up, he had resumed his previous level of activity without limitations or recurrent symptoms. Clinical examination demonstrated a stable knee with full range of motion. The postoperative Lysholm score was 100/100, indicating an excellent functional outcome.

## Discussion

Meniscal preservation has become a fundamental principle in contemporary knee surgery because of the crucial role of the meniscus in load transmission, shock absorption, joint stability, and cartilage protection [1,2]. Numerous studies have demonstrated that meniscectomy is associated with an increased risk of degenerative changes and osteoarthritis, particularly in young and active patients [3,4]. Consequently, whenever feasible, repair should be preferred over meniscal resection.

Despite improvements in arthroscopic techniques, failure after primary meniscal repair remains a recognized challenge [3]. Management of failed meniscal repair remains controversial, with some surgeons favoring partial meniscectomy, whereas others advocate revision repair in an attempt to preserve meniscal tissue [4].

The present case is of particular interest because the index lesion was a bucket-handle tear of the medial meniscus, a lesion commonly considered suitable for repair in young and active patients. Despite primary arthroscopic repair and appropriate rehabilitation, healing failure occurred. In such situations, revision repair may provide an alternative to meniscectomy, allowing preservation of meniscal tissue and maintenance of normal knee biomechanics.

In the present case, revision repair was selected because of the patient's young age, functional demands, and the desire to preserve meniscal function. Arthroscopic exploration demonstrated a persistent bucket-handle tear of the medial meniscus located within the red-white zone, consistent with failure of the primary repair. This region has limited but potentially sufficient healing capacity when combined with biological stimulation [1,2].

Biological augmentation techniques have gained increasing interest in recent years [5-11]. Among these strategies, autologous fibrin clot augmentation represents a simple, low-cost, and readily available option. Fibrin clots contain platelets, growth factors, and cytokines that may promote cellular migration, angiogenesis, and tissue healing [5,6].

Several authors have reported favorable outcomes following fibrin clot augmentation in primary meniscal repair, including complex and high-risk meniscal tears. The biological properties of fibrin clot augmentation may be particularly relevant in revision settings, where the healing potential of previously repaired tissue may be compromised [5,8,11]. More recently, Davies et al. reported low clinical failure rates in high-risk meniscal repairs augmented with fibrin clot [8]. Furthermore, recent systematic reviews have highlighted the growing interest in fibrin clot augmentation while emphasizing the need for higher-quality evidence [7].

A notable strength of this report is the comprehensive surgical documentation, including preoperative magnetic resonance imaging, arthroscopic visualization of the lesion, preparation of the fibrin clot, clot implantation, and the final repair construct. Furthermore, the patient achieved complete symptom resolution and returned to both work and sports activities one year after surgery, with an excellent postoperative Lysholm score of 100/100.

The main limitations of this report are its single-patient design, the absence of a documented preoperative Lysholm score preventing direct comparison of functional outcomes before and after revision surgery, the relatively limited follow-up duration of one year, and the absence of postoperative MRI confirmation of healing. Nevertheless, the excellent clinical outcome observed at one year suggests that revision repair augmented with an autologous fibrin clot may represent a valuable meniscus-preserving strategy after failure of primary repair of a bucket-handle medial meniscal tear. Structural healing, however, could not be confirmed because postoperative MRI or second-look arthroscopy was not performed [7-11].

Further studies involving larger patient cohorts and longer follow-up periods are required to determine the long-term effectiveness of fibrin clot augmentation in revision meniscal repair.

## Conclusions

Revision repair augmented with an autologous fibrin clot may represent a valuable meniscus-preserving option after failure of primary repair of a bucket-handle medial meniscal tear. In the present case, this technique resulted in complete symptom resolution, restoration of knee function, and successful return to both work and sports activities at one-year follow-up. Although structural healing could not be confirmed in the absence of postoperative MRI or second-look arthroscopy, the excellent clinical outcome observed supports further investigation of this biological augmentation strategy in selected revision cases. Further studies involving larger patient populations and longer follow-up periods are needed to better define its role in revision meniscal repair.
